# Supplementary material for: Chiral metal-organic frameworks incorporating nanozymes as neuroinflammation inhibitors for managing Parkinson’s disease
Source: Nat Commun. 2023 Dec 8;14:8137. doi: 10.1038/s41467-023-43870-3 (PMC10709450; doi:10.1038/s41467-023-43870-3)
Supplement: Supplementary file 1 — Supplementary Information [file 41467_2023_43870_MOESM1_ESM.docx]

**Supplementary Information**

**Chiral metal-organic frameworks incorporating nanozymes as neuroinflammation inhibitors** **for managing Parkinson’s disease**

*Wei Jiang ^1, 3, #^, Qing Li ^1, #,^ *, Ruofei Zhang ^4^, Jianru Li ^4^, Qianyu Lin ^1^, Jingyun Li ^3, 4^, Xinyao Zhou ^2^, Xiyun Yan,^3, 4,^ * Kelong Fan ^3, 4,^ **

^1^Application Center for Precision Medicine, the Second Affiliated Hospital of Zhengzhou University, Henan 450052, China.

^2^School of Engineering and Applied Science, University of Pennsylvania, Philadelphia, 19104, USA.

^3^Nanozyme Medical Center, Academy of Medical Sciences, Zhengzhou University, Zhengzhou 450001, China.

^4^CAS Engineering Laboratory for Nanozyme, Key Laboratory of Biomacromolecules, Institute of Biophysics, Chinese Academy of Sciences, 15 Datun Road, Beijing 100101, China.

*Corresponding authors: [lq1515012032@163.com](mailto:lq1515012032@163.com) (Q. Li), [yanxy@ibp.ac.cn](mailto:yanxy@ibp.ac.cn) (X. Yan), and [fankelong@ibp.ac.cn](mailto:fankelong@ibp.ac.cn) (K. Fan).

# These authors contributed equally to this work.

The supplementary information includes:

*1. Supplementary Methods*

*2. Supplementary Figures (1-23)*

*3. Supplementary Tables (1-2)*

**Supplementary Methods**

**Materials.** All reagents and solvents were used as received, unless otherwise indicated. Zinc nitrate hexahydrate, 2-methylimidazole, PVP (K12), PVP (K16), PVP (K30), PVP (K90), and H_2_PtCl_6_ were purchased from Sigma-Aldrich. Hexadecyl trimethyl ammonium bromide (CTAB), L-histidine, D-histidine, and other chiral amino acids were purchased from Adamas. DMEM and FBS were purchased from Gibco. SH-SY5Y cells were purchased from Pricella (CL-0208). BEnd.3 cells were purchased from Pricella (CL-0598). Thiazolyl blue tetrazolium bromide (MTT) was purchased from Sigma-Aldrich. Endocytosis inhibitors including sodium azide (NaN_3_), chlorpromazine (CPZ), dynasore, sucrose and Methyl-β-cyclodextrin (MβCD) were all purchased from Sigma-Aldrich.

**Characterizations.** The morphology of Ptzyme, Ptzyme@D-ZIF, and Ptzyme@L-ZIF were analyzed by TEM (HT7700, Hitachi, Japan). The hydrodynamic size and zeta potential of nanozymes were determined by Nanosizer Pro (Malvern, Britain). ESR spectroscopy was carried out by an ESR spectrometer (A300-10/12, Bruker, Germany) and DMPO was used as a superoxide radical and hydroxyl radical catching agent. Ultraviolet-visible spectroscopy (UV-vis) was performed on a Lengguang UV670 spectrophotometer. Fourier transform infrared spectra (FT-IR) was obtained on a Thermo Scientific Nicolet iS20 spectrometer. Powder X-ray diffraction (PXRD) patterns were collected on a D8 ADVANCE X-ray powder diffractometer (Bruker, Germany) with Cu Kα radiation (λ = 1.5406 Å), in which data were collected from 5 to 45^o^ at a scan rate of 15^o^ min^-1^. Circular Dichroism (CD) spectrum was carried out on a Chirascan Plus Circular Dichroism spectrometers (Applied Photophysics Ltd, England), in which the nanozymes solution at a concentration of 0.5 mg mL^-1^ in water were used as test samples.

**Synthesis of Ptzyme.** In a typical procedure, 62.5 mg PVP (K30) powders were first dissolved in 90 mL methanol under stirring, and then a 6.0 mM H_2_PtCl_6_ aqueous solution (10 mL) was added dropwise, followed by continuously stirring for 5 min. After being heated to reflux for 3 h, the PVP-stabilized Ptzyme were formed. Then rotary evaporation was utilized to remove methanol and water. After collecting and washing over 10 times to remove excess free PVP, the obtained samples were dried by vacuum.

**SOD-like activity of nanozymes.** The O_2_^•−^ elimination rates of nanozymes were used to calculate their SOD-like activity. Cytochrome C was used as an indicator for O_2_^•−^, which was produced by the xanthine-xanthine oxidase (X-XOD) system. The control system consisted of 0.5 mL xanthine, 0.5 mL cytochrome C, 0.2 mL xanthine oxidase, and 1.8 mL PBS buffer. The O_2_^•−^ concentration of the control system was determined by measuring the increase in absorbance at 550 nm (ΔA1) of cytochrome C using an ultraviolet spectrophotometer (TuxiT6, Beijing) for 1 minute. After adjusting ΔA1 to 0.025, nanozymes with different concentrations (0.000417 ⁓ 0.125 mg mL^-1^) were individually added to the control system, and the increase in absorbance at 550 nm (ΔA2) of cytochrome C was recorded for 1 minute. The O_2_^•−^ elimination rate of nanozymes was calculated using the formula: (ΔA1 - ΔA2) / ΔA1 * 100%.

**CAT-like activity of nanozymes.** The CAT-like activity of nanozymes was determined by measuring the increase in O_2_ concentration in a 0.3% H_2_O_2_ solution using a Dissolved Oxygen Meter (InPro 6860i, Mettler Toledo, Switzerland). The reaction system comprised of 200 μL nanozyme (40 μg mL^-1^) and 0.3% H_2_O_2_ in PBS buffer (pH 7.4) or acetate buffer (pH 4.5). The decomposition of H_2_O_2_ by nanozymes was assessed by monitoring the decrease in absorbance at 240 nm of H_2_O_2_.

**POD- and OXD-like activities of nanozymes.** The POD- and OXD-like activities of nanozymes were measured by monitoring the increase in the absorbance at 652 nm of oxidized TMB using a microplate reader (Spark, Tecan, Austria). For POD detection, the reaction system (100 µL) contains 10 µL of nanozyme (0.1 mg mL^-1^), 5 µL of H_2_O_2_ (30%), 1 µL of TMB (20 mg mL^-1^), 84 µL of PBS buffer (pH 7.4) or acetate buffer (pH 4.5). For OXD detection, the reaction system (100 µL) contains 10 µL of nanozyme, 1 µL of TMB (20 mg mL^-1^), 89 µL of PBS buffer (pH 7.4) or acetate buffer (pH 4.5).

**ABTS^•+^ scavenging capacity of nanozymes**. The ABTS^•+^ scavenging activities of nanozymes were measured as previously described^1^. The ABTS reagent was prepared by reacting the ABTS stock solution (5 mM) with a solution of potassium persulfate (K_2_S_2_O_8_, final concentration 2.45 mM) to facilitate the generation of radicals. The resulting solution was stored in the dark for 16 h. Then, the solution was diluted with a 5 mM phosphate-buffered saline (PBS) buffer (pH 7.4) to obtain an absorbance of 0.70 ± 0.05 at 734 nm, which was measured using a spectrophotometer. For the determination of antioxidant activity, 1 mL of ABTS reagent was mixed with 100 μL of either nanozyme samples (1 mg mL^-1^) or PBS buffer. The absorbance was then continuously measured at 734 nm for 10 mins.

**Construction and Treatment of Parkinson’s Disease (PD) Model.** Before the investigation, the mice were subjected to rotarod performance on the rotation rod and those exhibited behavioral consistencies were selected for subsequent studies. Mice were subjected to intraperitoneal MPTP administration (30 mg kg^-1^ day^-1^ for consecutive 7 days) to induce a PD-like phenotype and were randomly divided into 4 groups (n = 6 for each group): control (healthy mice), MPTP, MPTP + Ptzyme@D-ZIF and MPTP + Ptzyme@L-ZIF. Ptzyme@D-ZIFs and Ptzyme@L-ZIFs (5 mg kg^-1^ day^-1^) were intravenously injected into mice every other day for 5 times after MPTP injection. MPTP and control groups received saline only. The behavior tests were carried out on the fifth day after the final intravenous injection and then all mice were sacrificed for the subsequent studies.

**Brain tissue distribution.** PD mice were injected with Ptzyme@D-ZIFs and Ptzyme@L-ZIFs (5 mg kg^-1^) through the tail vein (n = 6 for each group). 24 h after the administration, three mice in each group the mice were sacrificed and organs including heart, liver, spleen, lung, kidney and brain were collected. Then, the tissues were weighed and homogenized to get the percentage of injected dose per gram of tissue (% ID g^-1^) by ICP-MS (Agilent 7800, China). In addition, the rest three mice in each group were sacrificed for brain tissue collection. The collected tissues were fixed with 2.5% glutaraldehyde for 24 h and subjected to TEM (Hitachi H-7650, Japan) observation.

**Quantitative real-time PCR (qPCR).** Total RNAs of the brain tissue (n = 3) were extracted by Trizol Reagent and cDNA was generated by PrimeScript^®^ RT reagent Kit (RR047A, TaKaRa, Japan). Afterward, the expression levels of *TNF-α*, *IL-6,* and *IL-1β* were detected on 7500 Real-Time PCR System (Applied Biosystems, Foster City, Canada) with TB Green Premix Ex Taq^TM^ Ⅱ kit (RR820A, Takara, Japan). The data were calculated by the 2^−ΔΔCT^ method and *β-actin* was used as a control gene for normalization. Primer sequences used were as follows:

*TNF-α* forward: 5′-ATCCGCGACGTGGAACTG-3′;

reverse: 5′-ACCGCCTGGAGTTCTGGAA -3′;

*IL-6* forward: 5′-GAGGATACCACTCCCAACAGACC -3′;

reverse: 5′-AAGTGCATCATCGTTGTTCATACA -3′;

*IL-1β* forward: 5′-GAGCACCTTCTTTTCCTTCATCTT -3′;

reverse: 5′-TCACACACCAGCAGGTTATCATC -3′.

*β-actin* forward: 5′-GCTCTGGCTCCTAGCACCAT -3′;

reverse: 5′-GCCACCGATCCACACACAGAGT -3′.

**Transcriptome analysis.** Total RNA in brain SNpc was prepared using Trizol Reagent (Invitrogen, USA) and Oligo (dT) magnetic beads were applied to enrich mRNA with polyA structure among total RNA. All RNA was interrupted to fragments in 200 ~ 300 bp by ion-interruption. The first cDNA strand was synthesized with random primers with 6-base and reverse transcriptase with RNA as a template, and the second cDNA strand was synthesized with the first cDNA strand as a template. After the library was constructed, PCR amplification was used to enrich the library fragments, and then the library was selected according to the fragment size, which was 450 bp. Then, the total concentration and effective concentration of the library were detected through 2100 Bioanalyzer (Agilent, USA). After RNA extraction, purification, and library construction, the library was subjected to paired-end sequencing using the Next-Generation Sequencing (NGS) based on Illumina HiSeq X10 (Illumina, San Diego, USA).

For bioinformatics analysis, HTSeq (<https://www.huber.embl.de/users/anders/HTSeq/doc/overview.html>) was used to compare the Read Count value of genes, and then standardized by the fragments per kilobase of exon per million mapped (FPKM) method. DEGs were identified using DESeq (<http://www.bioconductor.org/packages/release/bioc/html/DESeq.html>) (fold change >1 and *P* value < 0.05). The volcano map of differentially expressed genes was plotted by ggplots2 (<http://ggplot2.org/>) software package in R language. TopGO (http://www.bioconductor.org/packages/release/bioc/html/RamiGO.html) was used for GO enrichment analysis. During the analysis, the gene list and gene number of each term were calculated by the differential genes annotated by [Blast2go](https://www.blast2go.com/) (https://www.blast2go.com/), and then *P* values were calculated by hypergeometric distribution method (*P* value < 0.05 was the standard for significant enrichment) to find out the GO term in which the differential genes were significantly enriched, thus determining the main biological functions of differential genes. Besides, KEGG enrichment analysis was carried out and then annotated by KAAS (<https://www.genome.jp/tools/kaas/>) (*P* value < 0.05 compared with the whole-transcriptome background was the standard for significant enrichment).

**Detection of ROS and MDA.** Levels of ROS were determined according to the manufacturer’s protocol of the assay kit (E004, Nanjing Jiancheng Bioengineering Institute, Nanjing, China). Briefly, the brain tissues (n = 3) were mechanically prepared into single-cell suspension and collected by centrifugation at 500 *g* for 15 min. The cells were diluted into 1.0 × 10^6^ per sample. Afterward, 10 μM DCFH-DA was added and incubated at room temperature for 60 min. The fluorescence of cells was detected at an excitation wavelength of 485 nm by the Infinite F200 Pro microplate reader (TECAN, Switzerland) and ROS levels were expressed by the fluorescence intensity.

The brain tissues of mice (n = 3) were obtained following treatments and then homogenized in physiological saline at 4°C. After centrifugation, the supernatant was used for BCA detection and MDA content analysis using a commercial assay kit (A003, Nanjing Jiancheng Bioengineering Institute, China) in accordance with the manufacturer’s protocol.

**Cell viability assay.** 96-well plates were seeded with SH-SY5Y cells, and the cell density was maintained at around 7,000 cells well^-1^. Various nanozyme-integrated chiral ZIFs with different concentrations (20 ⁓ 100 mg mL^-1^) were introduced into the cells for 6 h. A subsequent addition of 2 mM MPP^+^ was incubated for 24 h. Subsequently, 20 μL of MTT solution (5 mg mL^-1^) was added to each well and incubated for an additional 4 h in the cell incubator. Finally, dimethyl sulfoxide (DMSO) was added to each well, and the absorbance (492 nm) was measured by a GF-M3000 microplate reader (CAIHONG, Shandong, China).

**ROS detection of SH-SY5Y cells.** ROS contents in SH-SY5Y cells were determined according to the Reactive Oxygen Species Assay Kit (Beyotime, Nanjing, China). Briefly, cells were seeded into 6-well plates with densities of 2.0 × 10^5^ cells per well and pre-treated with Ptzyme@D-ZIFs and Ptzyme@L-ZIFs (80 μg mL^-1^) for 6 h. Cells were then exposed to 2 mM MPP^+^ for 2 h at 37°C and stained with 10 μM DCFH-DA for 30 min at 37°C. ROS expression was detected by Olympus IX73P1F fluorescence microscopy (Tokyo, Japan) for intuitive observation and quantitative detection by flow cytometer (Accuri C6, Beckman, NJ), followed by analysis through Beckman Coulter software.

**Cell apoptosis analysis.** SH-SY5Y cells were inoculated into plates with densities of 1.5 × 10^5^ cells per well and cultured overnight. Subsequently, cells were incubated with Ptzyme@D-ZIFs and Ptzyme@L-ZIFs (80 μg mL^-1^) for 6 h followed by treatment with 2 mM MPP^+^ for 24 h, after which the cells were harvested and subjected to Annexin V-FITC/PI staining referred to the manufacture’s protocol (Bestbio, Shanghai, China). The apoptosis of SH-SY5Y cells was detected by flow cytometer and analyzed using Beckman Coulter software. The caspase-3/7 activities were measured using the caspase-3/7 activity assay kits (Promega, Madison, WI, USA) and detected by the TECAN Infinite F200 Pro Multi Detection Plate Reader (Mannedorf, Switzerland).

**Intracellular MDA contents detection.** SH-SY5Y cells were inoculated into plates with densities of 1.5×10^5^ cells per well and cultured overnight. Subsequently, cells were incubated with Ptzyme@D-ZIFs and Ptzyme@L-ZIFs (80 μg mL^-1^) for 6 h and then treated with 2 mM MPP^+^ for 24 h. Intracellular MDA contents were determined according to the manufacturer’s protocol of the assay kit (A003-4-1, Nanjing Jiancheng Bioengineering Institute, Nanjing, China). The supernatant was discarded and the cells were scraped off with a cell scraper and transferred to the centrifuge tube. 0.5 mL extracting solution was added and mixed for two minutes. Afterward, the cells were broken by ultrasonic method taken 0.1 mL into a new centrifuge tube, and then other reagents were added as required. Finally, after being taken out, the mixture was arranged for reaction at 95℃ for 40 min. After centrifugation (1500 *g*, 10 min), absorbance (530 nm) of the supernatant was measured via a microplate reader.

**Mitochondrial membrane potential detection.** SH-SY5Y cells were inoculated into plates with densities of 1.5 × 10^5^ cells per well and cultured overnight. Subsequently, cells were incubated with Ptzyme@D-ZIFs and Ptzyme@L-ZIFs (80 μg mL^-1^) for 6 h and then treated with 2 mM MPP^+^ for 24 h. and the mitochondrial membrane potential was measured with a JC-1 probe. Briefly, the treated cells were incubated with a JC-1 probe in the detection kit at room temperature for 40 min. Then, after washing with PBS, the mitochondrial membrane potentials were detected by fluorescence microscopy.

**Biosafety evaluation.** To evaluate the potential toxicity of various the nanozyme-integrated chiral ZIFs on major organs of mice, nanozyme-integrated chiral ZIFs (5 mg kg^-1^ day^-1^) or saline (control group) were intravenously injected into healthy C57BL/6 male mice (five-week-old, n = 3 for each group) every other day for 5 times. In addition, MPTP (30 mg kg^-1^ day^-1^) were subjected to intraperitoneal administration into healthy C57BL/6 male mice (five-week-old, n = 3) for a consecutive period of 7 days. After these mice were sacrificed, main organs including heart, liver, spleen, lung, and kidney were gained and fixed in 4 % paraformaldehyde for 48 h. After water flushing, the tissues were subjected to gradient dehydration with ethanol solutions. Then the tissues were treated twice in xylene and the tissue blocks were embedded with melted paraffin wax. After slicing via a microtome (RM2016, Leica, China), and cytoplasm and nucleus were stained with hematoxylin and eosin (H&E), respectively. Finally, the slices were photographed by fluorescence microscope (XSP-C204, CIC, China).

Moreover, the blood samples of the PD mice with or without nanozyme-integrated chiral ZIFs treatment, and the control group (saline treatment), were all collected and allowed to stand at room temperature for 30 min, followed by centrifugation at 800 *g* for 10 min to obtain the supernatant serum samples, in which alanine transaminase (ALT), aspartate aminotransferase (AST), blood urea nitrogen (BUN) and creatinine (CRE) were detected for hematological biochemical analyses.

**Supplementary figures**





**Supplementary Figure 1.** XRD pattern of Ptzyme (K30), Ptzyme (K12), Ptzyme (K16), Ptzyme (K90).


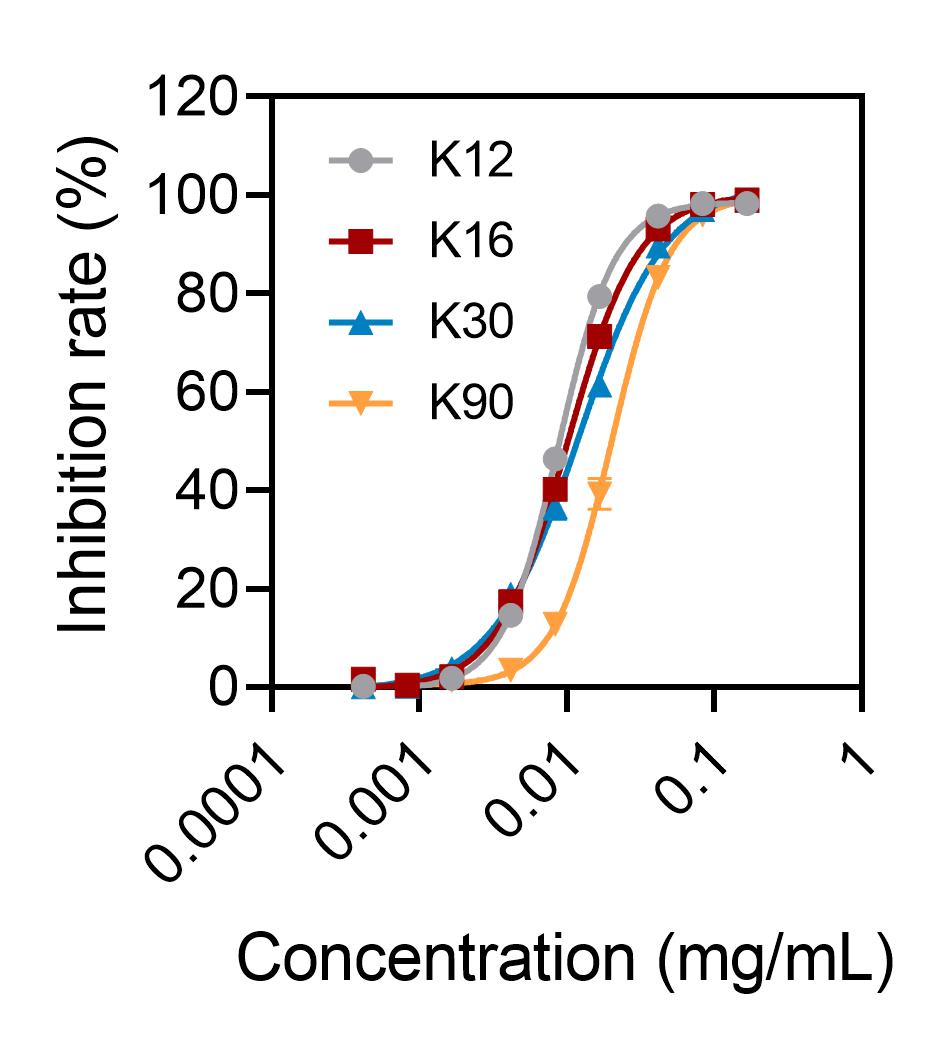


**Supplementary Figure 2.** The SOD-like activities of Ptzymes produced by using PVP with different molecular weights (K12, K16, K30, and K90), n = 3 independent experiments. Data represent the mean ± SD.


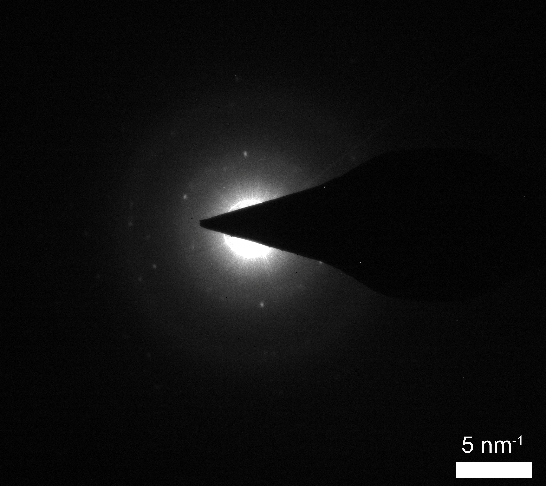


**Supplementary Figure 3.** SAED pattern of Ptzyme embedded in ZIF shell. The scale bar is 5 nm^-1^.


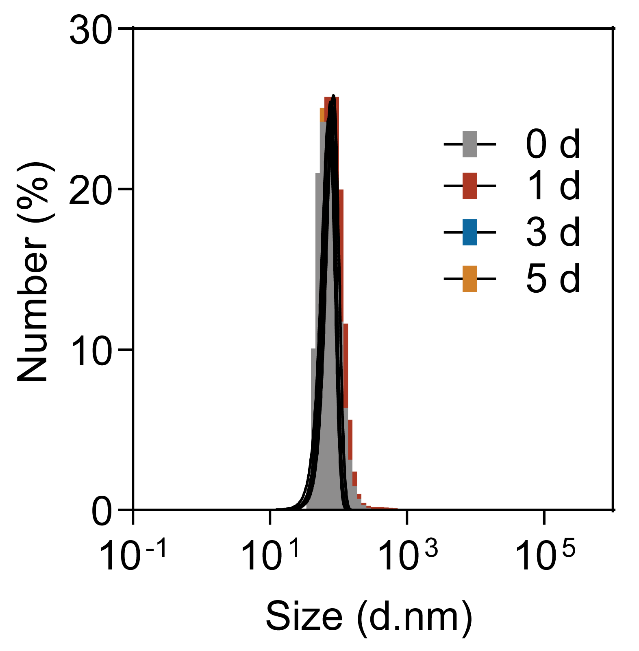


**Supplementary Figure 4.** Hydrodynamic diameter analyses of Ptzyme@ZIF upon dispersion in deionized water and after 5 days of storage.


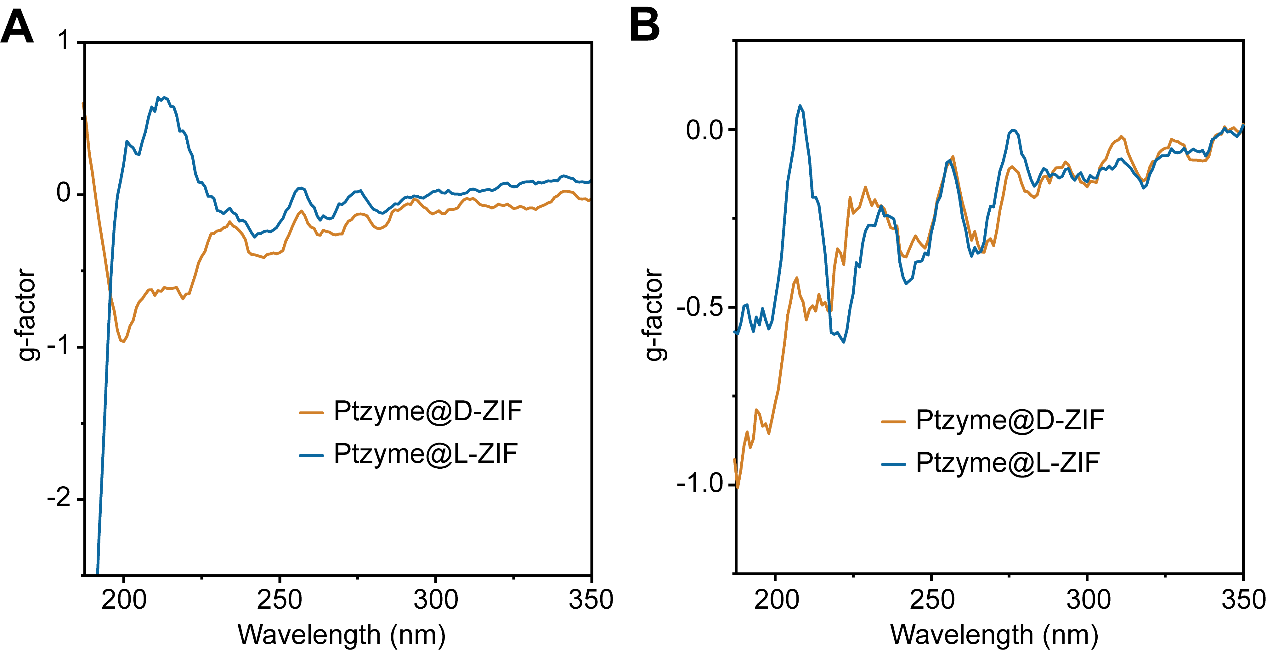


**Supplementary Figure 5.** Corresponding CD spectra of Ptzyme@L-ZIF and Ptzyme@D-ZIF produced by using D/L-Phenylalanine (A) or D/L-Methionine (B) as ligand.


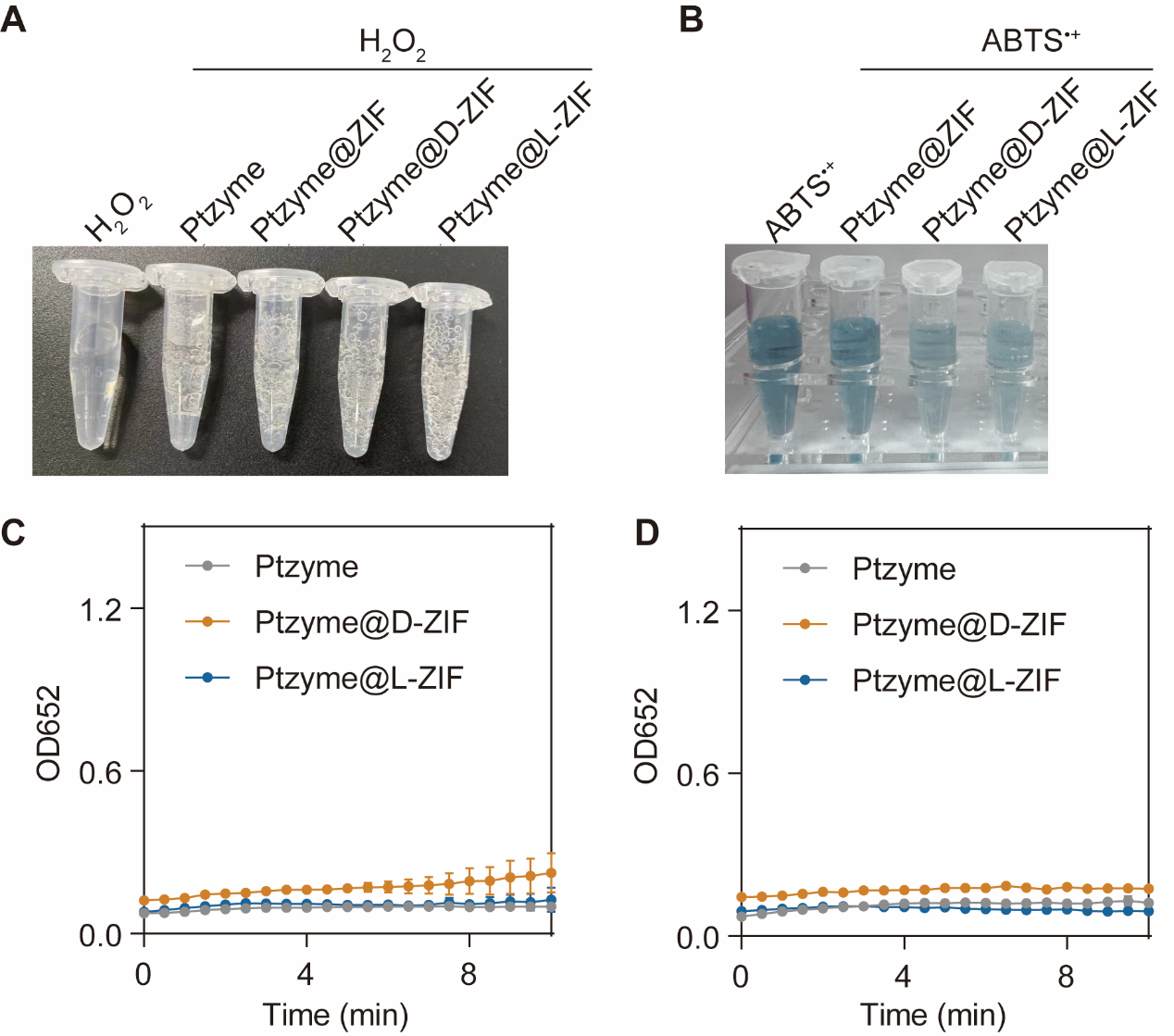


**Supplementary Figure 6.** The analyses of enzymatic activities of Ptzyme and nanozyme-integrated chiral ZIFs. (A) Representative O_2_ generation images of H_2_O_2_ catalyzed by Ptzyme@ZIF, Ptzyme@L-ZIF and Ptzyme@D-ZIF for 10 mins. (B) Representative images of ABTS^•+^ system catalyzed by Ptzyme@ZIF, Ptzyme@L-ZIF, and Ptzyme@D-ZIF for 8 mins. (C) Oxidase-like activities analyses of Ptzyme@ZIF, Ptzyme@L-ZIF and Ptzyme@D-ZIF at pH = 7.4, n = 3 independent experiments. Data represent the mean ± SD. (D) Peroxidase-like activities analyses of Ptzyme@ZIF, Ptzyme@L-ZIF and Ptzyme@D-ZIF at pH = 7.4, n = 3 independent experiments. Data represent the mean ± SD.


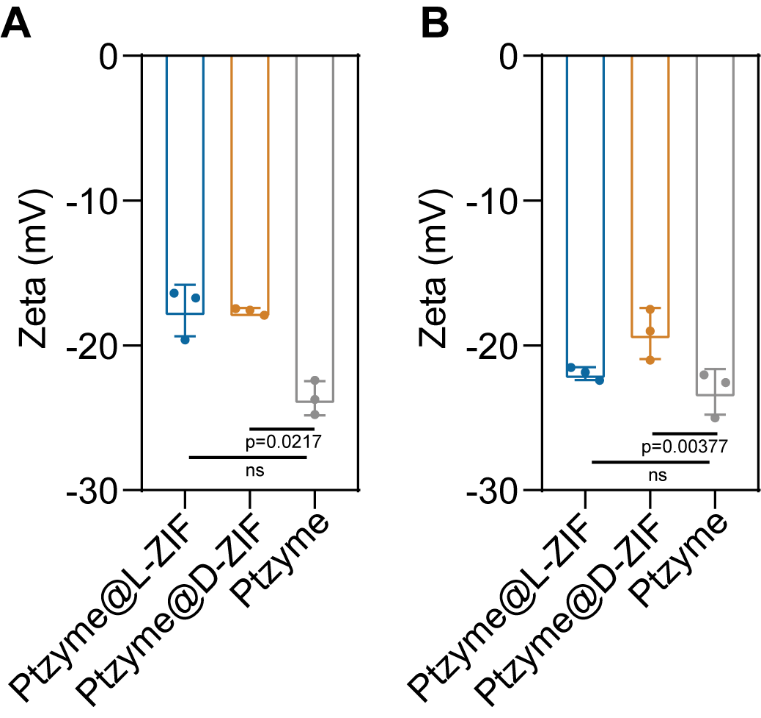


**Supplementary Figure 7.** The zeta potential analyses of Ptzyme, Ptzyme@D-ZIF, and Ptzyme@L-ZIF in different substrate solutions: (A) H_2_O_2_, and (B) H_2_O_2_ + ABTS, n = 3 independent experiments. Data represent the mean ± SD. The statistical analyses were conducted using GraphPad Prism 8.0.2. The outcomes were compared *via* one-way ANOVA (with Tukey’s post hoc correction for multiple comparisons). “ns” indicates not significant.


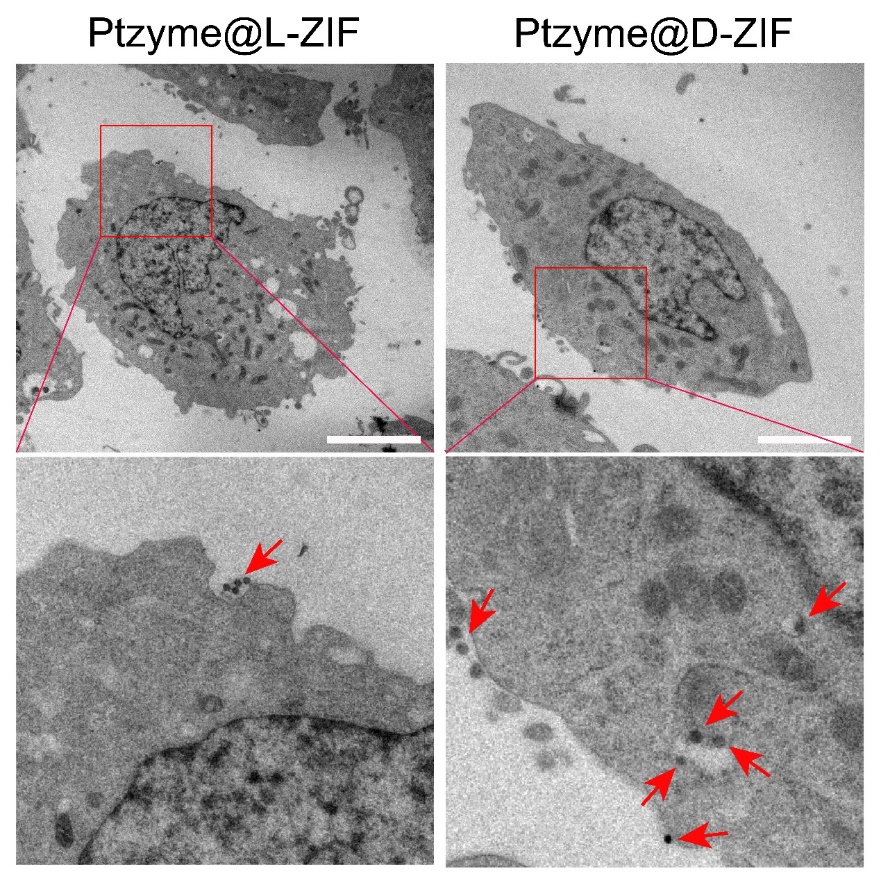


**Supplementary Figure 8.** TEM images of Ptzyme@L-ZIFs and Ptzyme@D-ZIFs entering into SHSY-5Y cells. The red arrows indicate the locations of the nanoparticles. The scale bars are 5 μm. A representative image of three biologically independent samples from each group is shown.


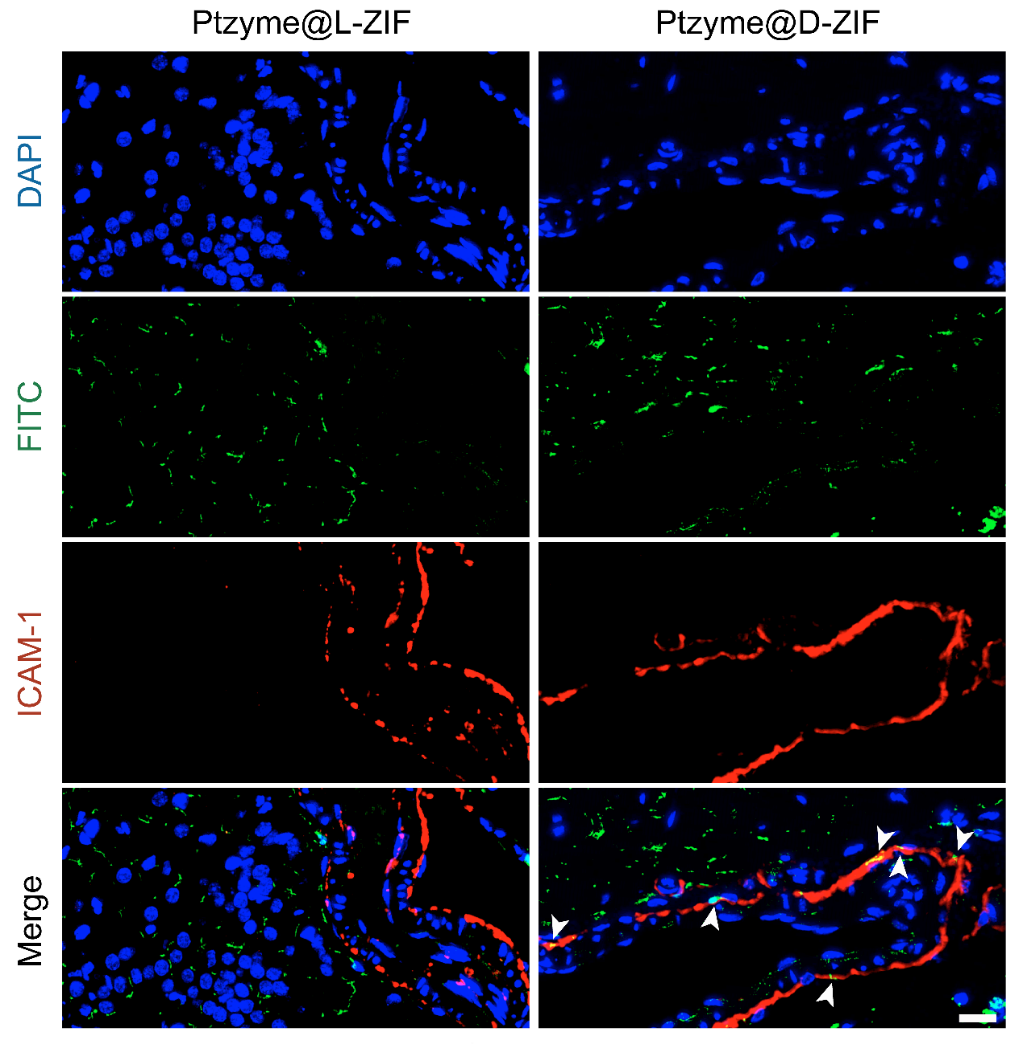


**Supplementary Figure 9.** The uptake of Ptzyme@L-ZIFs and Ptzyme@D-ZIFs into the brain endothelial cells of PD mice detected by immunohistochemical staining. The white arrows indicate the accumulations of Ptzyme@D-ZIFs in the brain endothelial vessels. The scale bar is 20 μm. A representative image of three biologically independent samples from each group is shown.


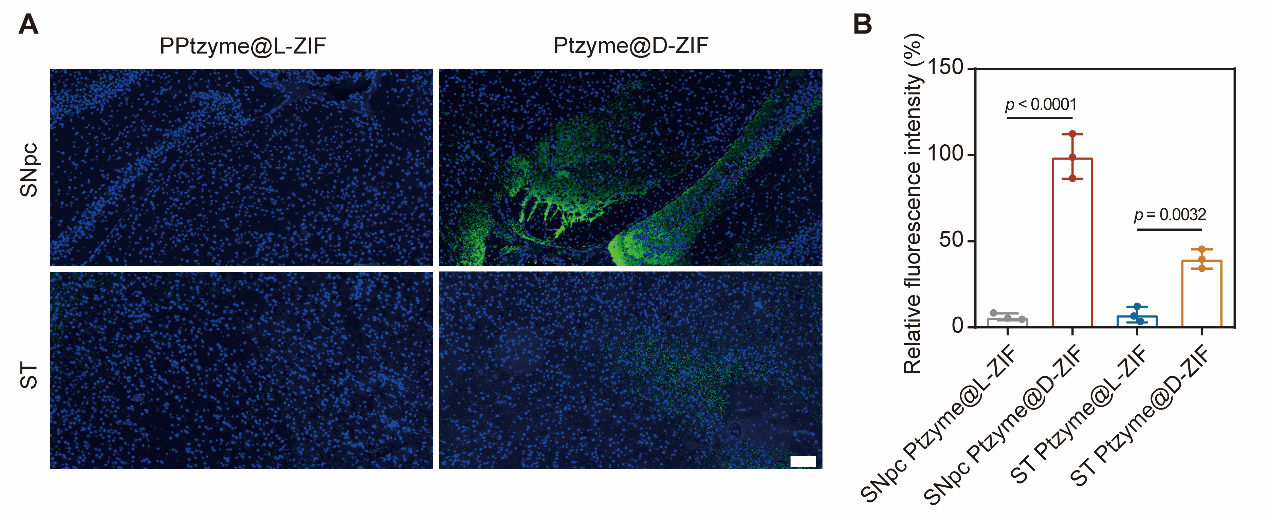


**Supplementary Figure 10.** The distribution of Ptzyme@L-ZIFs and Ptzyme@D-ZIFs in the SNpc and ST detected by immunofluorescence (A) and fluorescence quantification (B), n = 3 independent animals. Data represent the mean ± SD. The scale bar is 100 μm. A representative image of three biologically independent samples from each group is shown. The statistical analyses were conducted using GraphPad Prism 8.0.2. The outcomes were compared *via* one-way ANOVA (with Tukey’s post hoc correction for multiple comparisons).


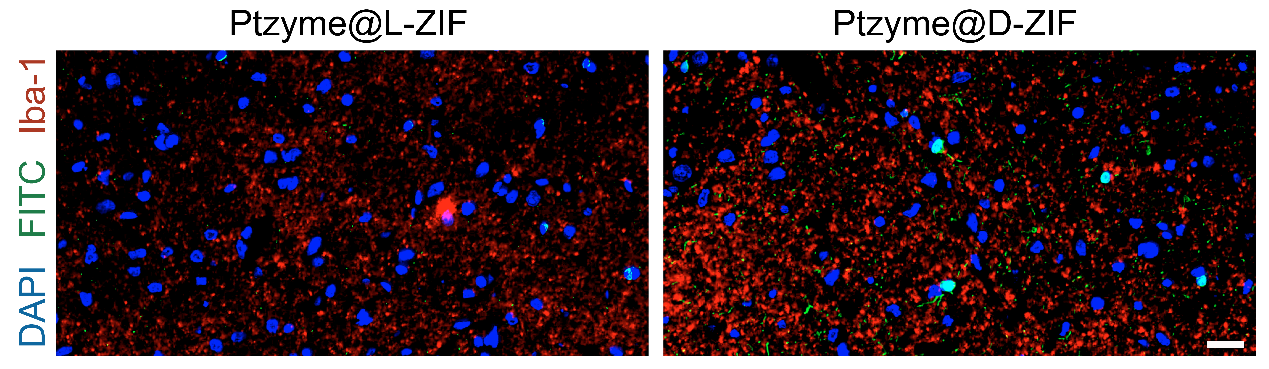


**Supplementary Figure 11.** The internalization of Ptzyme@L-ZIFs and Ptzyme@D-ZIFs in the glial cells of brains from PD mice detected by immunofluorescence. The scale bar is 20 μm. A representative image of three biologically independent samples from each group is shown.

**
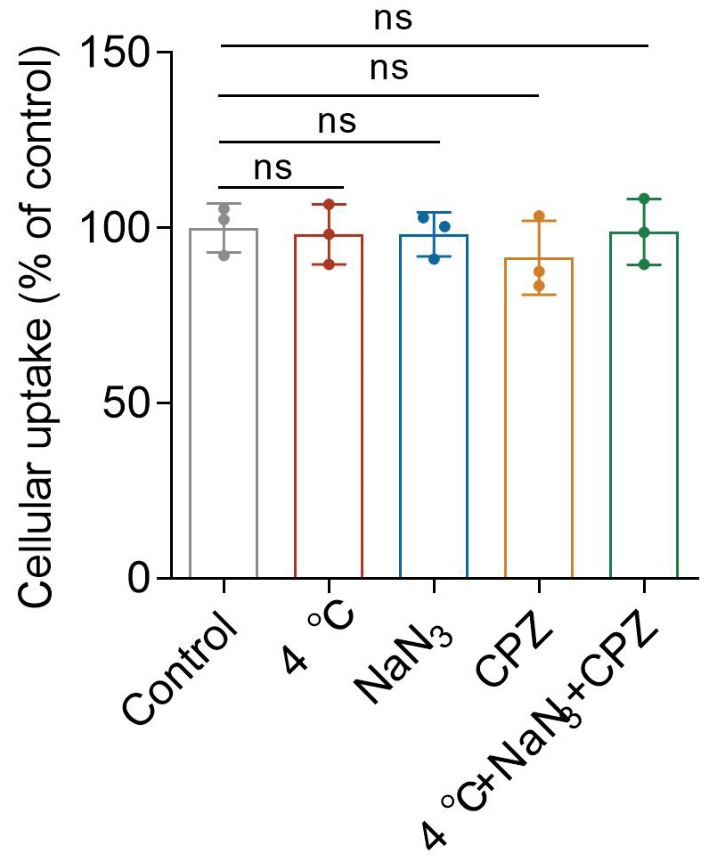
**

**Supplementary Figure 12.** The endocytic pathways of achiral Ptzyme@ZIFs analyzed by endocytosis inhibitors, n = 3 independent experiments. Data represent the mean ± SD. The statistical analyses were conducted using GraphPad Prism 8.0.2. The outcomes were compared *via* one-way ANOVA (with Tukey’s post hoc correction for multiple comparisons). “ns” indicates not significant.


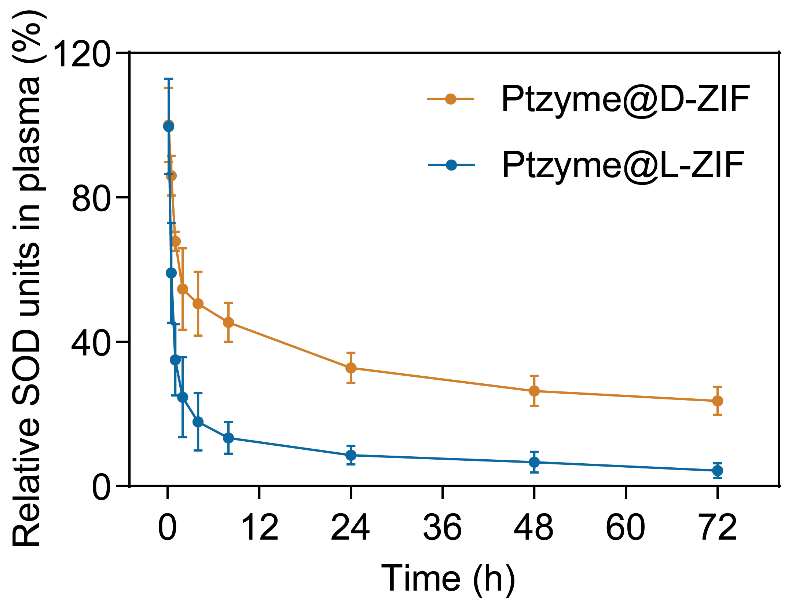


**Supplementary Figure 13.** The plasma levels of Ptzyme@D-ZIFs and Ptzyme@L-ZIFs detected by enzymatic activity after single *i.v.* injection, n = 3 independent animals. Data represent the mean ± SD.


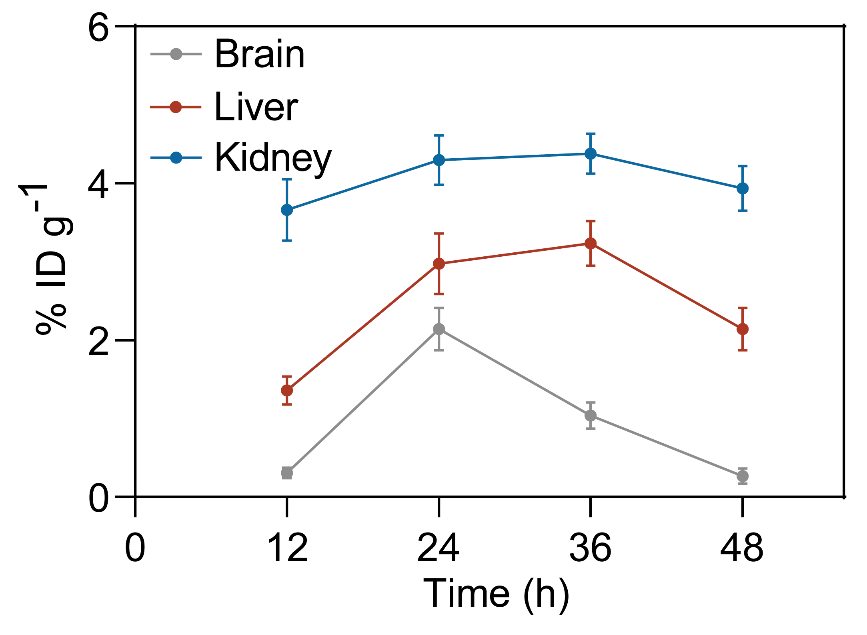


**Supplementary Figure 14.** ICP-MS analyses of the concentration of Ptzyme@D-ZIFs in the brain, as well as their metabolism processes through liver and kidney following a single intravenous injection, n = 3 independent animals. Data represent the mean ± SD.


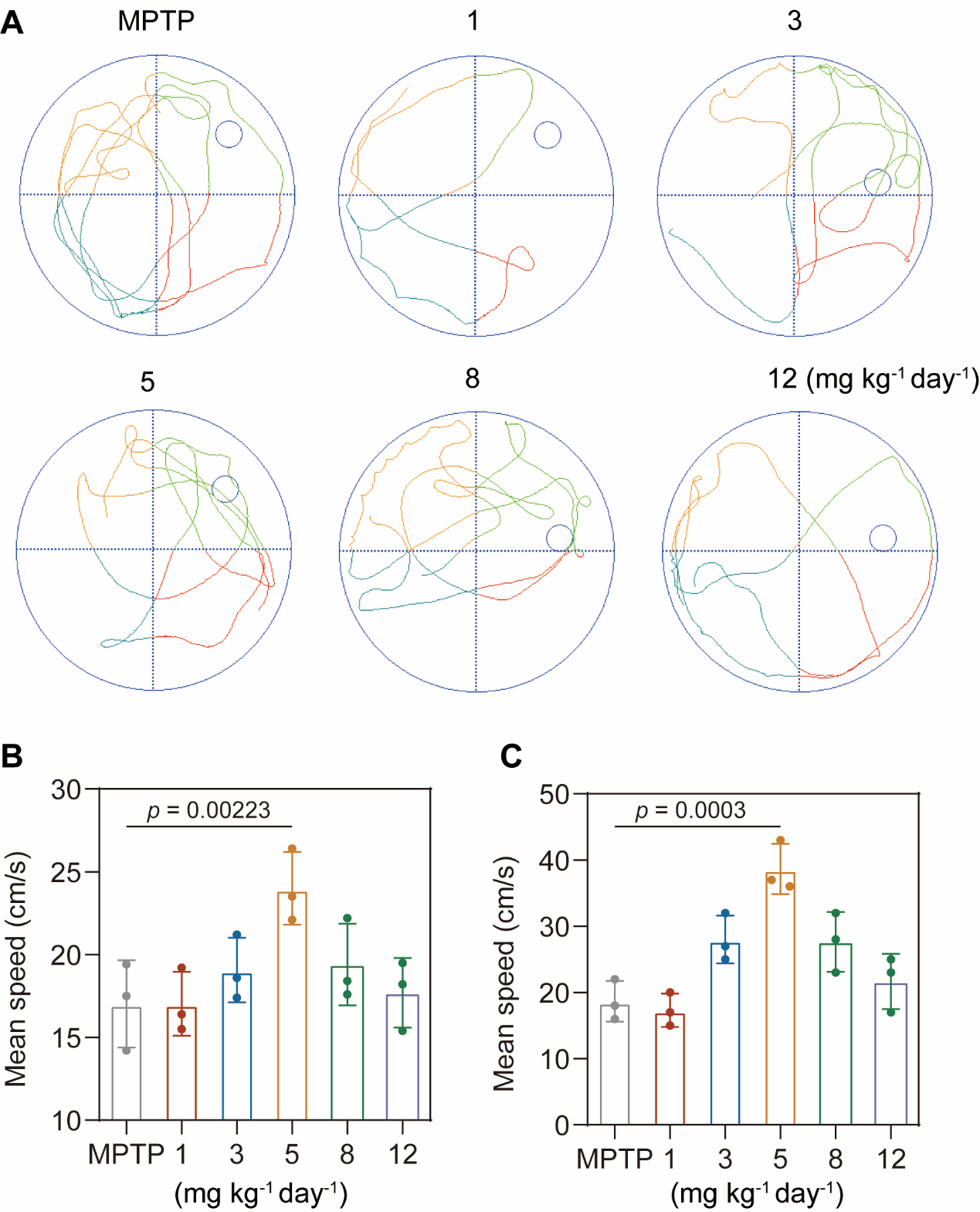


**Supplementary Figure 15.** Morris water maze test in the preliminary experiment with the administration concentration of Ptzyme@D-ZIFs varied from 1 to 12 mg kg^-1^ day^-1^. (A) The representative path tracing of mice, (B) the mean swimming speed of mice and (C) the relative time spent on the target quadrant, n = 3 independent animals. Data represent the mean ± SD. The statistical analyses were conducted using GraphPad Prism 8.0.2. The outcomes were compared *via* one-way ANOVA (with Tukey’s post hoc correction for multiple comparisons).


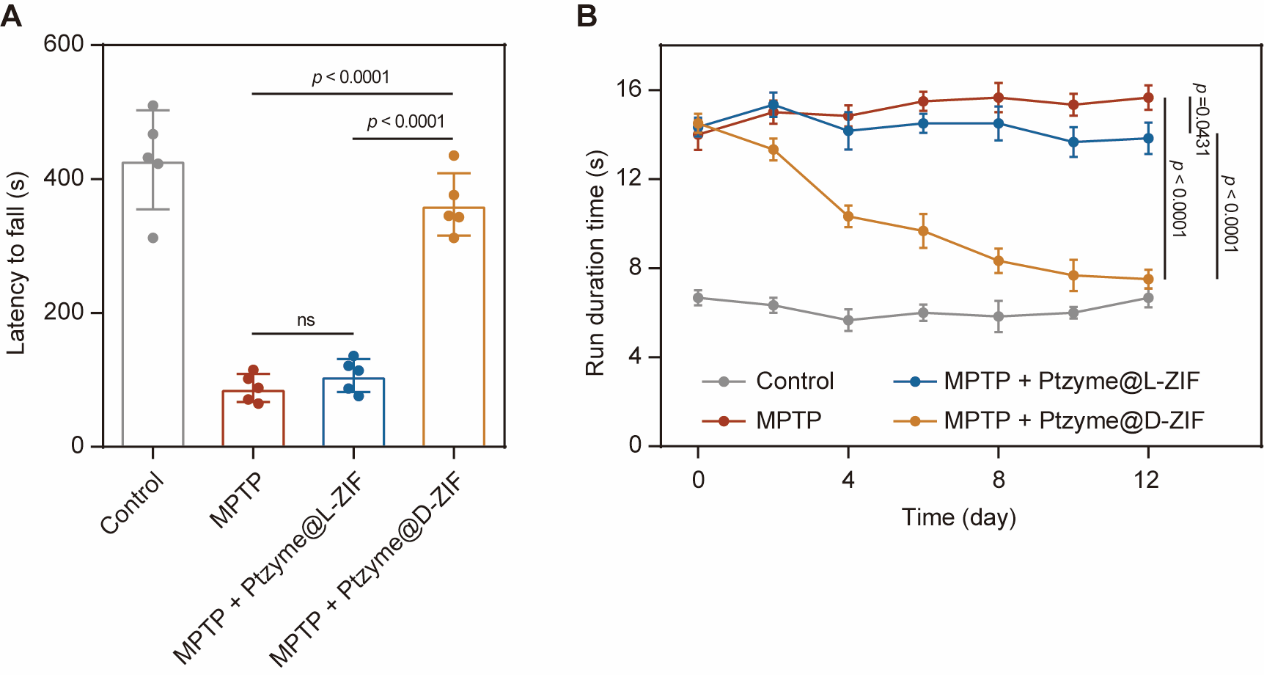


**Supplementary Figure 16.** Behavioral performance of PD mice after treatment with nanozyme-integrated chiral ZIFs detected by (A) rotarod test and (B) pole test, n = 6 independent animals. Data represent the mean ± SD. The statistical analyses were conducted using GraphPad Prism 8.0.2. The outcomes were compared *via* one-way ANOVA (with Tukey’s post hoc correction for multiple comparisons) in Supplementary Figure 16A and two-way ANOVA test (with Bonferroni’s multiple comparisons test) in Supplementary Figure 16B. “ns” indicates not significant.


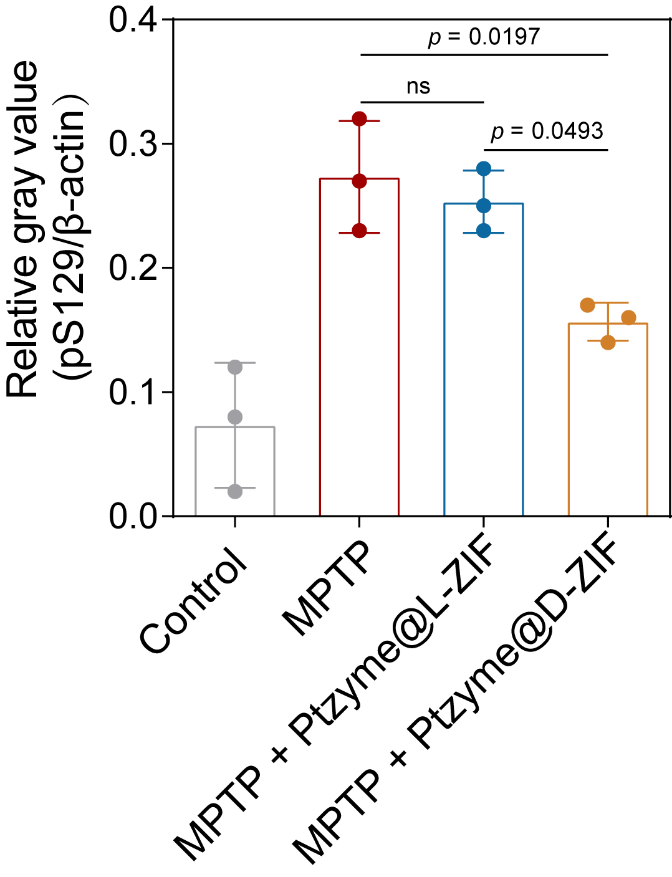


**Supplementary Figure 17.** Quantitative analysis of gray value of pS129 and β-actin**.** The statistical analyses were conducted using GraphPad Prism 8.0.2. The outcomes were compared *via* one-way ANOVA (with Tukey’s post hoc correction for multiple comparisons). n = 3 independent experiments. Data represent the mean ± SD. “ns” indicates not significant.


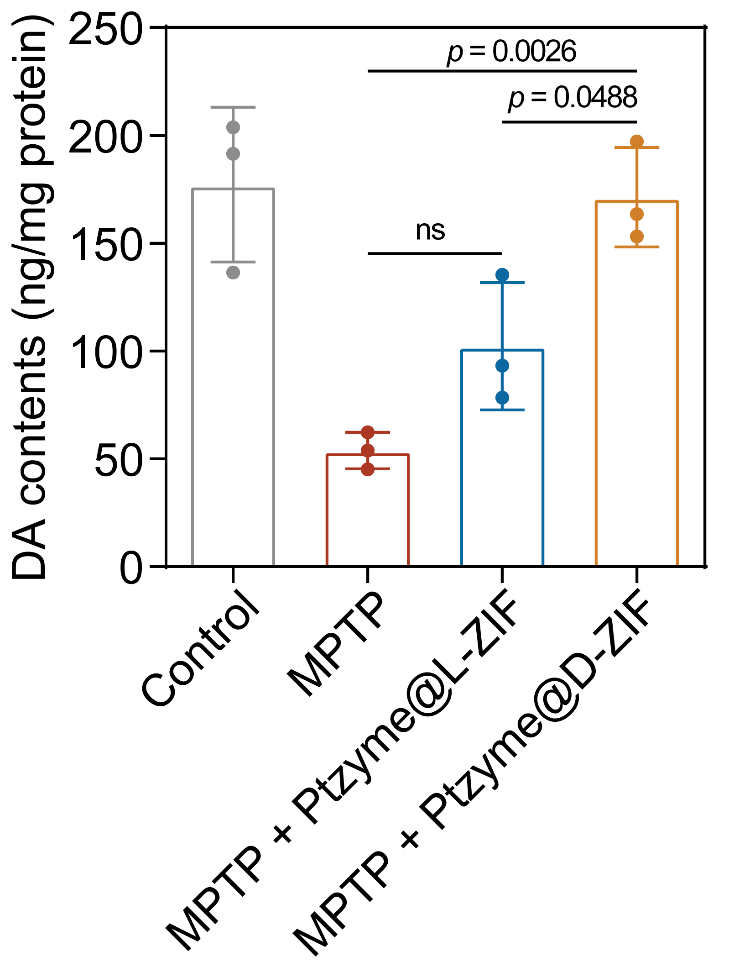


**Supplementary Figure 18.** Dopamine (DA) levels in the striatum of PD mice with or without nanozyme-integrated chiral ZIFs treatment, n = 3 independent animals. Data represent the mean ± SD. The statistical analyses were conducted using GraphPad Prism 8.0.2. The outcomes were compared *via* one-way ANOVA (with Tukey’s post hoc correction for multiple comparisons). “ns” indicates not significant.


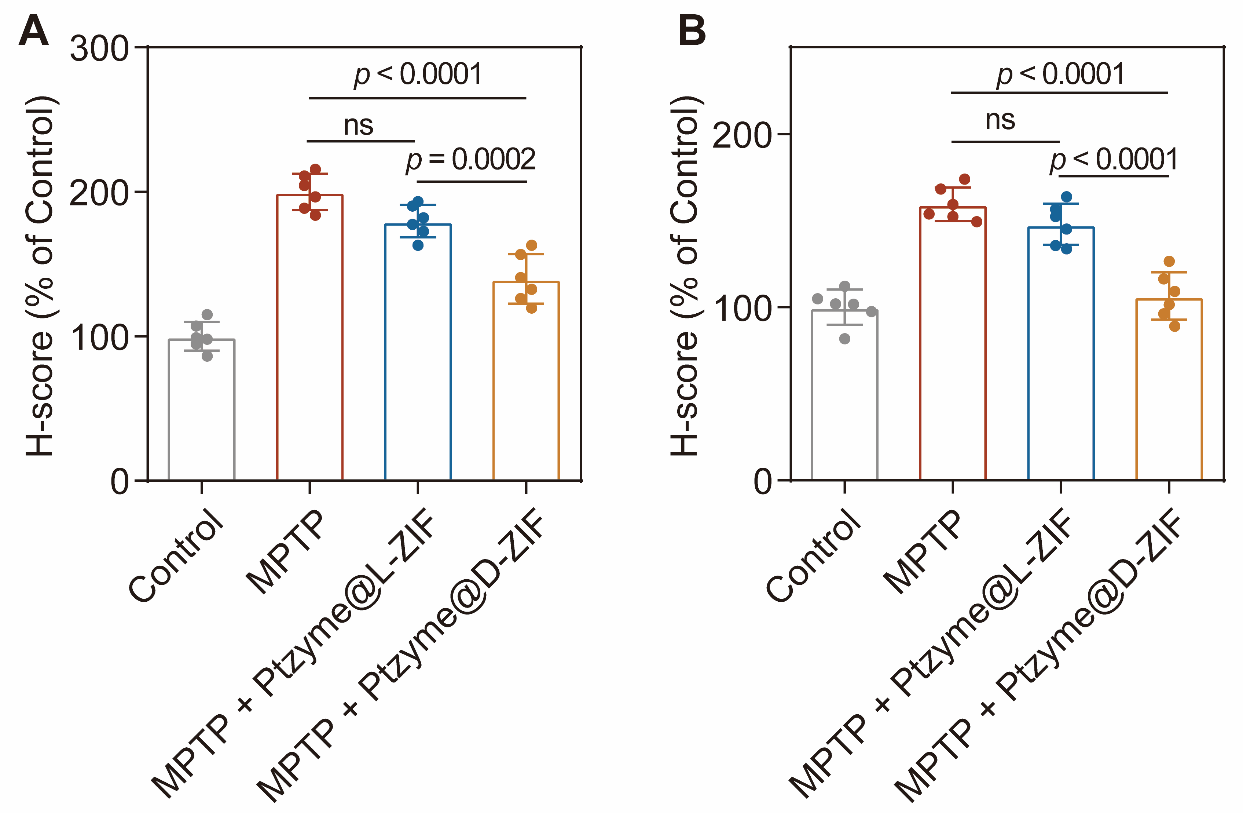


**Supplementary Figure 19.** Semi-quantitative analysis of GFAP (A) and Iba-1-positive (B) areas via the digital tissue section scanner and image analysis system, and expressed as the percentage of H-score of other groups to the control group, n = 6 independent animals. Data represent the mean ± SD. The statistical analyses were conducted using GraphPad Prism 8.0.2. The outcomes were compared *via* one-way ANOVA (with Tukey’s post hoc correction for multiple comparisons). “ns” indicates not significant.


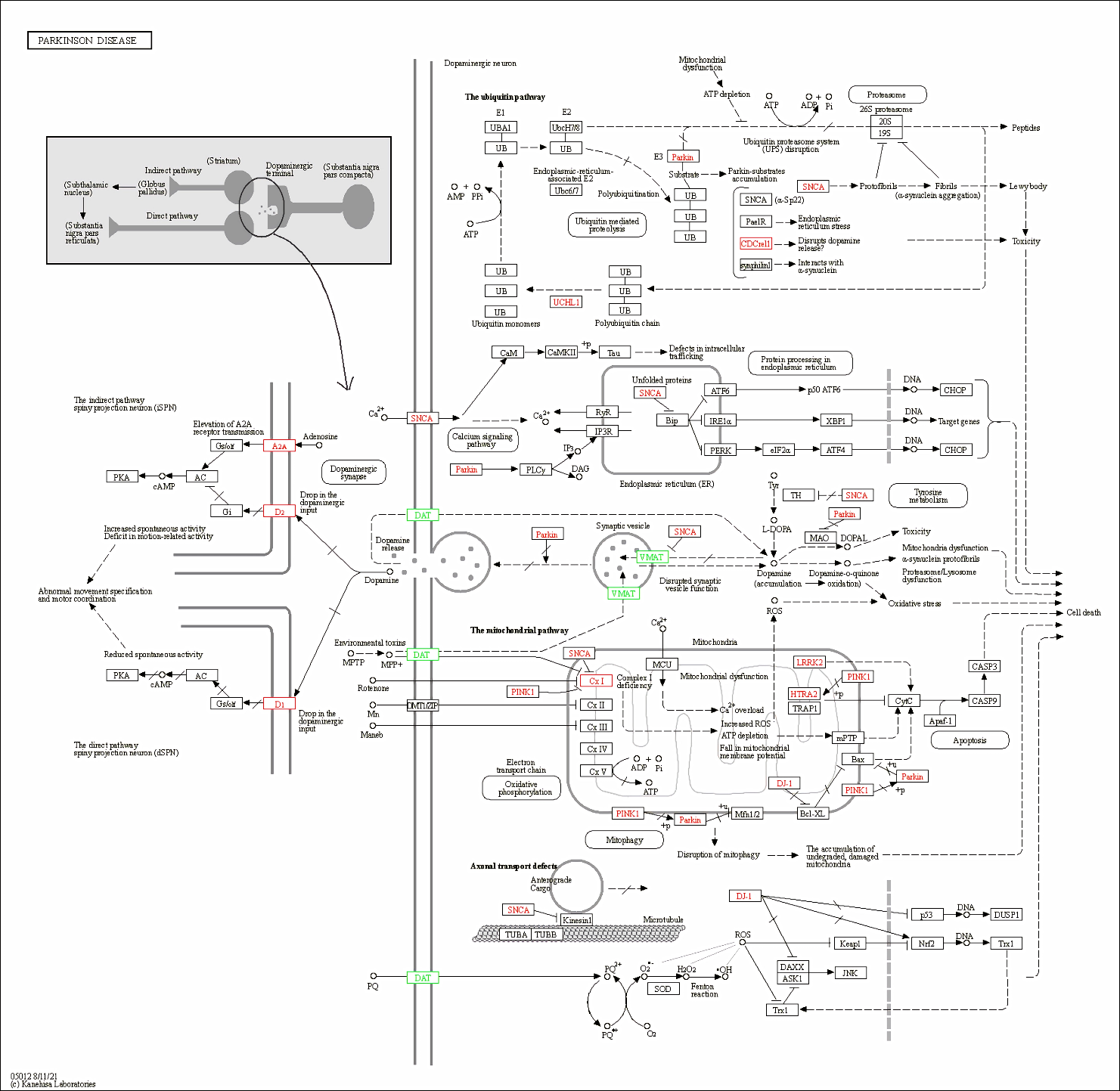
**Supplementary Figure 20.** KO analysis of PD mice with or without Ptzyme@D-ZIFs treatment.


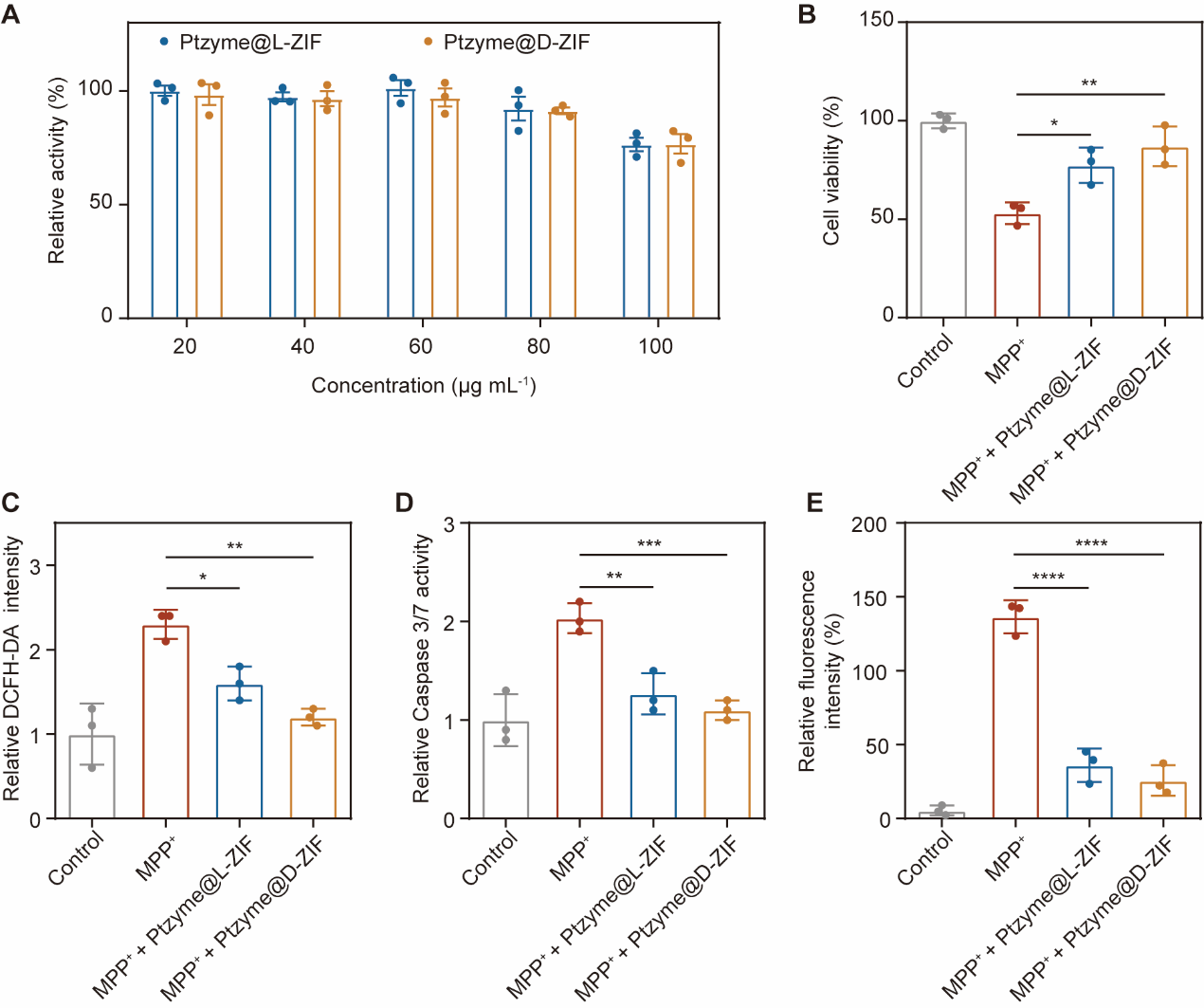


**Supplementary Figure 21.** *In vitro* analysis of the SH-SY5Y cells post-treatment with nanozyme-integrated chiral ZIFs. (A) Toxicity analyses of SH-SY5Y cells following treatment with nanozyme-integrated chiral ZIFs with concentrations varied from 20 to 100 μg/mL. (B) Viability of SH-SY5Y cells exposed to MPP^+^ after different treatments. (C) DCFH-DA fluorescence quantitative analysis showing the intracellular levels of ROS. (D) Prevention of MPP^+^ induced apoptosis by nanozyme-integrated chiral ZIFs analyzed through measuring of activity of effector caspase 3/7. (E) The quantitative result of mitochondrial membrane potential expressed as the fluorescence ratio of monomer to JC-1 aggregates, n = 3 independent experiments. Data represent the mean ± SD. The statistical analyses were conducted using GraphPad Prism 8.0.2. The outcomes were compared *via* one-way ANOVA (with Tukey’s post hoc correction for multiple comparisons). “ns” indicates not significant.


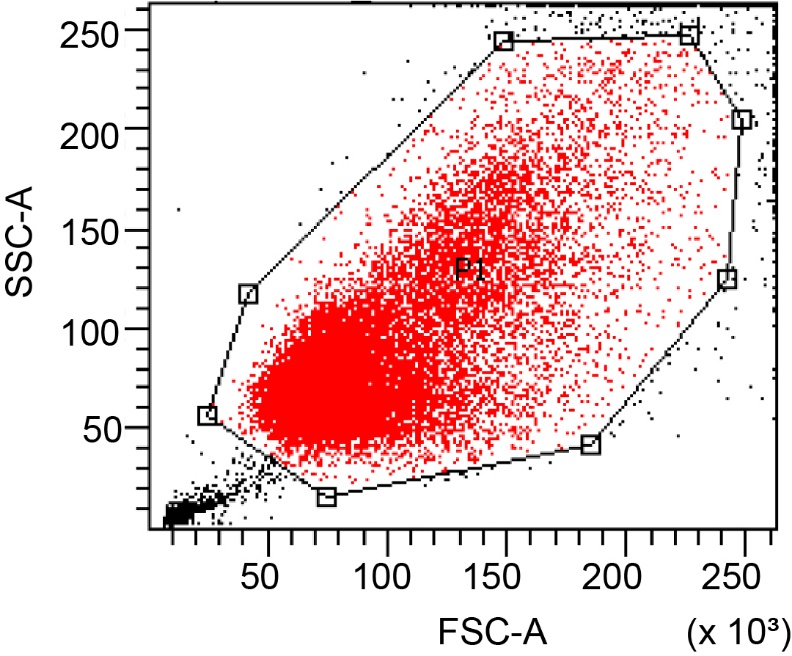


**Supplementary Figure 22.** Gating strategy to determine the cell apoptosis presented in Figure 8B.

**
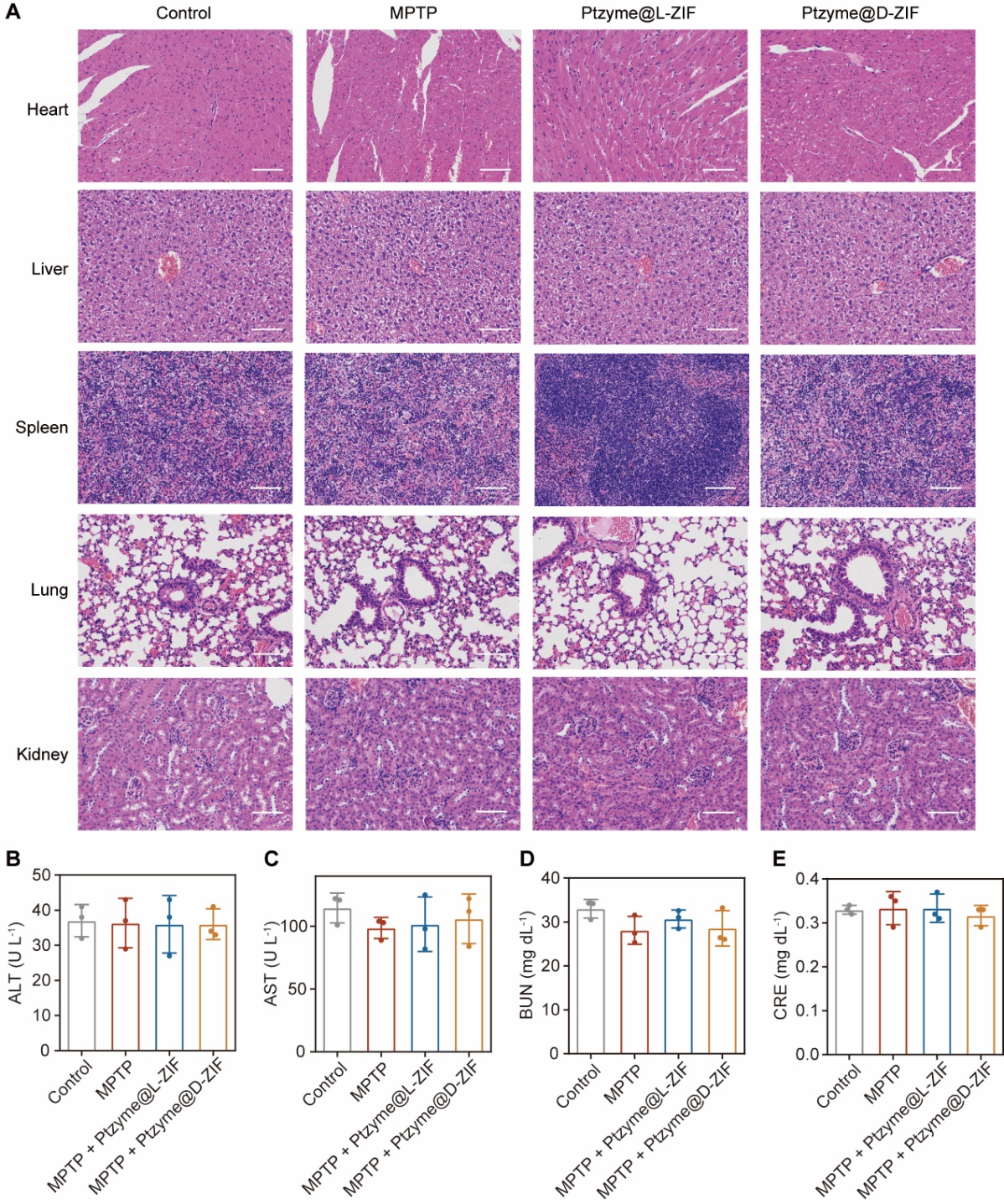
**

**Supplementary Figure 23.** The toxicity analyses of the major organs and serum parameters from the mice post-treatment with nanozyme-integrated chiral ZIFs. (A) Hematoxylin and eosin staining of the liver, lung, kidney, heart, and spleen in the mice treated with MPTP, Ptzyme@L-ZIFs or Ptzyme@D-ZIFs. The scale bars are 50 μm. A representative image of three biologically independent samples from each group is shown. (B) Serum biochemical analyses of the MPTP-induced PD model mice treated with Ptzyme@L-ZIFs or Ptzyme@D-ZIFs. ALT, alanine transaminase; AST, aspartate transaminase (C); BUN, blood urea nitrogen (D); CRE, creatinine (E), n = 3 independent animals. Data represent the mean ± SD.

**Supplementary Tables**

**Supplementary Table 1.** Biological process analysis of PD mice with or without Ptzyme@D-ZIFs treatment.

| Terms | Up | Down | DEG | Total | Representative up genes | Representative down genes |
| --- | --- | --- | --- | --- | --- | --- |
| Regulation of biological quality | 65 | 69 | 134 | 3925 | ENSMUSG00000000531(Tamalin), ENSMUSG00000005583(Mef2c), ENSMUSG00000007207(Stx1a), ENSMUSG00000007617(Homer1), ENSMUSG00000019935(Slc17a8) | ENSMUSG00000000125(Wnt3),ENSMUSG00000000263(Glra1),ENSMUSG00000003657(Calb2),ENSMUSG00000004035(Gstm7) |
| Trans-synaptic signaling | 26 | 26 | 52 | 706 | ENSMUSG00000005583(Mef2c), ENSMUSG00000007207(Stx1a), ENSMUSG00000007617(Homer1), ENSMUSG00000019935(Slc17a8), ENSMUSG00000020178(Adora2a) | ENSMUSG00000000263(Glra1),ENSMUSG00000003657(Calb2),ENSMUSG00000010825(Grid2ip),ENSMUSG00000021609(Slc6a3) |
| Nervous system development | 43 | 52 | 95 | 2401 | ENSMUSG00000001827(Folr1), ENSMUSG00000003282(Plag1), ENSMUSG00000005583(Mef2c), ENSMUSG00000015843(Rxrg), ENSMUSG00000017491(Rarb) | ENSMUSG00000000125(Wnt3),ENSMUSG00000001504(Irx2),ENSMUSG00000004872(Pax3),ENSMUSG00000010175(Prox1) |

**Supplementary Table 2.** Molecular function ontology analysis of PD mice with or without Ptzyme@D-ZIFs treatment.

| Terms | Up | Down | DEG | Total | Representative up genes | Representative down genes |
| --- | --- | --- | --- | --- | --- | --- |
| Ion transmembrane transport | 32 | 23 | 55 | 1137 | ENSMUSG00000001827(Folr1),  ENSMUSG00000005583(Mef2c),  ENSMUSG00000007617(Homer1),  ENSMUSG00000019935(Slc17a8),  ENSMUSG00000020599(Rgs9) | ENSMUSG00000000263(Glra1),  ENSMUSG00000004035(Gstm7),ENSMUSG00000021609(Slc6a3),ENSMUSG00000021948(Prkcd) |
| Ion channel activity | 15 | 14 | 29 | 419 | ENSMUSG00000022342(Kcnv1), ENSMUSG00000025221(Kcnip2), ENSMUSG00000028033(Kcnq5), ENSMUSG00000030592(Ryr1), ENSMUSG00000033007(Asic4) | ENSMUSG00000000263(Glra1),  ENSMUSG00000027577(Chrna4)，ENSMUSG00000028532(Cachd1),ENSMUSG00000031344(Gabrq) |

**References**

1. Nenadis, N.; Wang, L.F.; Tsimidou, M.; Zhang, H.Y. Estimation of scavenging activity of phenolic compounds using the ABTS^•+^ assay. J Agric Food Chem 2004;52: 4669e74.
