# Supplementary material for: Chiral metal-organic frameworks incorporating nanozymes as neuroinflammation inhibitors for managing Parkinson’s disease
Source: Nat Commun. 2023 Dec 8;14:8137. doi: 10.1038/s41467-023-43870-3 (PMC10709450; doi:10.1038/s41467-023-43870-3)
Supplement: Supplementary file 3 — Reporting Summary [file 41467_2023_43870_MOESM3_ESM.pdf]

nature portfolio

Corresponding author(s): Kelong Fan

Last updated by author(s): Nov 21, 2023

Reporting Summary

Nature Portfolio wishes to improve the reproducibility of the work that we publish. This form provides structure for consistency and transparency in reporting. For further information on Nature Portfolio policies, see our Editorial Policies and the Editorial Policy Checklist.

Statistics

For all statistical analyses, confirm that the following items are present in the figure legend, table legend, main text, or Methods section.

n/a	Confirmed	
☐	☒	The exact sample size (*n*) for each experimental group/condition, given as a discrete number and unit of measurement
☐	☒	A statement on whether measurements were taken from distinct samples or whether the same sample was measured repeatedly
☐	☒	The statistical test(s) used AND whether they are one- or two-sided<br>*Only common tests should be described solely by name; describe more complex techniques in the Methods section.*
☐	☒	A description of all covariates tested
☐	☒	A description of any assumptions or corrections, such as tests of normality and adjustment for multiple comparisons
☐	☒	A full description of the statistical parameters including central tendency (e.g. means) or other basic estimates (e.g. regression coefficient) AND variation (e.g. standard deviation) or associated estimates of uncertainty (e.g. confidence intervals)
☐	☒	For null hypothesis testing, the test statistic (e.g. *F*, *t*, *r*) with confidence intervals, effect sizes, degrees of freedom and *P* value noted<br>*Give P values as exact values whenever suitable.*
☒	☐	For Bayesian analysis, information on the choice of priors and Markov chain Monte Carlo settings
☒	☐	For hierarchical and complex designs, identification of the appropriate level for tests and full reporting of outcomes
☒	☐	Estimates of effect sizes (e.g. Cohen's *d*, Pearson's *r*), indicating how they were calculated

Our web collection on statistics for biologists contains articles on many of the points above.

Software and code

Policy information about availability of computer code

Data collection	The morphology of Ptzyme, Ptzyme@D-ZIF, and Ptzyme@L-ZIF were observed by TEM (HT7700, Hitachi, Japan). The hydrate particle size and zeta potential of nanozymes were determined by Nanosizer Pro (Malvern, Britain). ESR spectroscopy was carried out by an ESR spectrometer (A300-10/12, Bruker, Germany) and DMPO was used as a superoxide radical and hydroxyl radical catching agent. Ultraviolet-visible spectroscopy (UV-vis) was performed on a Lengguang UV670 spectrophotometer. Fourier transform infrared spectra (FT-IR) was obtained on a Thermo Scientific Nicolet iS20 spectrometer. Powder X-ray diffraction (PXRD) patterns were collected on a D8 ADVANCE X-ray powder diffractometer (Bruker, Germany), in which data were collected from 5 to 45o at a scan rate of 15o/min. Circular Dichroism (CD) spectrum was carried out on a Chirascan Plus Circular Dichroism spectrometers (Applied Photophysics Ltd, England).
Data analysis	The RNA analysis for transcriptomics was subjected to paired-end sequencing using the Next-Generation Sequencing (NGS) based on Illumina HiSeq X10 (Illumina, San Diego, USA). For bioinformatics analysis, HTSeq (https://www.huber.embl.de/users/anders/HTSeq/doc/overview.html) was used to compare the Read Count value of genes, and then standardized by the fragments per kilobase of exon per million mapped (FPKM) method. DEGs were identified using DESeq (http://www.bioconductor.org/packages/release/bioc/html/DESeq.html) . The volcano map of differentially expressed genes was plotted by ggplots2 (http://ggplot2.org/) software package in R language. TopGO (http://www.bioconductor.org/packages/release/bioc/html/RamiGO.html) was used for GO enrichment analysis. During the analysis, the gene list and gene number of each term were calculated by the differential genes annotated by Blast2go (https://www.blast2go.com/). KEGG enrichment analysis was carried out and then annotated by KAAS (https://www.genome.jp/tools/kaas/). Other data were analyzed using Flowjo V10 and Graphpad Prism (8.0.2) software.

For manuscripts utilizing custom algorithms or software that are central to the research but not yet described in published literature, software must be made available to editors and reviewers. We strongly encourage code deposition in a community repository (e.g. GitHub). See the Nature Portfolio guidelines for submitting code & software for further information.

Data

Policy information about availability of data

All manuscripts must include a data availability statement. This statement should provide the following information, where applicable:

- Accession codes, unique identifiers, or web links for publicly available datasets
- A description of any restrictions on data availability
- For clinical datasets or third party data, please ensure that the statement adheres to our policy

All data needed to evaluate the conclusions in the paper are present in the paper and/or the Supplementary Information and Source Data. The accession code of National Center for Biotechnology Information concerning the raw sequence reads in RNA-seq data is PRJNA1032155. Additional data is available from the corresponding authors upon request. Source data are provided with this paper.

Research involving human participants, their data, or biological material

Policy information about studies with human participants or human data. See also policy information about sex, gender (identity/presentation), and sexual orientation and race, ethnicity and racism.

Reporting on sex and gender	No relevant studies were included in our manuscript.
Reporting on race, ethnicity, or other socially relevant groupings	No relevant studies were included in our manuscript.
Population characteristics	No relevant studies were included in our manuscript.
Recruitment	No relevant studies were included in our manuscript.
Ethics oversight	No relevant studies were included in our manuscript.

Note that full information on the approval of the study protocol must also be provided in the manuscript.

Field-specific reporting

Please select the one below that is the best fit for your research. If you are not sure, read the appropriate sections before making your selection.

☒ Life sciences ☐ Behavioural & social sciences ☐ Ecological, evolutionary & environmental sciences

For a reference copy of the document with all sections, see nature.com/documents/nr-reporting-summary-flat.pdf

Life sciences study design

All studies must disclose on these points even when the disclosure is negative.

Sample size	For experiments involving enzyme-like assay, in vitro cell experiments, biodistribution, biosafety analyses, behavioral evaluation in replication experiment, and therapeutic related experiments in PD mice, n=3 was chosen as the minimal replicate numbers. For experiments involving pathological evaluations and behavioral evaluation, n=6 was chosen as the minimal replication numbers. The sample sizes employed in this study are consistent with previously published works (Nature Communications, 2023, 14:2661; Adv. Mater. 2022, 34, 2108435).
Data exclusions	No data was excluded in this study.
Replication	Our experiments were repeated for three times or six times, and we confirm that all attempts at replication were successful.
Randomization	All allocations were random.
Blinding	The investigations were blinded to group allocation during data collection and analysis.

Reporting for specific materials, systems and methods

We require information from authors about some types of materials, experimental systems and methods used in many studies. Here, indicate whether each material, system or method listed is relevant to your study. If you are not sure if a list item applies to your research, read the appropriate section before selecting a response.

Materials & experimental systems

n/a	Involved in the study	
	x	Antibodies
	x	Eukaryotic cell lines
x		Palaeontology and archaeology
	x	Animals and other organisms
x		Clinical data
x		Dual use research of concern
x		Plants

Methods

n/a	Involved in the study	
x		ChIP-seq
	x	Flow cytometry
x		MRI-based neuroimaging

Antibodies

Antibodies used: Antibodies against TH (GB12181, Servicebio, China, dilution 1:500), pS129 (ab51253, Abcam, China, clone EP1536Y, dilution 1:500), GFAP (GB12096, Servicebio, China, dilution 1:1000), IBA-1 (GB13105-1, Servicebio, China, dilution 1:1000), GPX4 (GPX4, ab125066, Abcam, clone EPNCIR144, dilution 1:1000).

Validation:
TH:https://www.servicebio.cn/search-result?search=GB12181
GFAP:https://www.servicebio.cn/search-result?search=GB12096
IBA-1:https://www.servicebio.cn/search-result?search=GB13105-1
pS129:https://www.abcam.cn/products/primary-antibodies/alpha-synuclein-phospho-s129-antibody-ep1536y-ab51253.html
GPX4:https://www.abcam.cn/products/primary-antibodies/glutathione-peroxidase-4-antibody-epncir144-ab125066.html

Eukaryotic cell lines

Policy information about cell lines and Sex and Gender in Research

Cell line source(s): SHSY-5Y cells were purchased from Pricella (CL-0208). BEnd.3 cells were purchased from Pricella (CL-0598). The sex was not considered in the study.

Authentication: DNA was extracted from appropriate amount of cells using Chelex×100, and 20 STR loci and sex identification loci were amplified using 21 Cellid System. PCR products were detected using ABI3130×1 genetic analyzer, and the detection results were analyzed using GeneMapper IDX software. DAMZ, JCRB, Cellosaurus and other databases.

Mycoplasma contamination: The cell lines were tested negative for mycoplasma contamination.

Commonly misidentified lines (See ICLAC register): The cell models used in this study belongs to mature technology, and the cell models are against the list of known misidentified cell lines maintained by the International Cell Line Authentication Committee.

Animals and other research organisms

Policy information about studies involving animals; ARRIVE guidelines recommended for reporting animal research, and Sex and Gender in Research

Laboratory animals: C57BL/6 male mice of five-week-old are housed in a specific pathogen free-grade animal facility with air humidity 40%-70%, ambient temperature (22-24 ℃), and 12-h dark/12-h light cycle.

Wild animals: We didn't use wild animals in our study.

Reporting on sex: All animal experiments were conducted using male mice, as only male C57BL/6 mice have been reported to be sensitive to MPTP modeling. All animal studies and analyses in this study were based on male mice.

Field-collected samples: No field collected samples were used in the study.

Ethics oversight: All of the animal experiments were approved by the Institution Animal Ethics Committee of Zhengzhou University (license No. ZZU-LAC20210625[07]).

Note that full information on the approval of the study protocol must also be provided in the manuscript.

Flow Cytometry

Plots

Confirm that:

☒ The axis labels state the marker and fluorochrome used (e.g. CD4-FITC).

☒ The axis scales are clearly visible. Include numbers along axes only for bottom left plot of group (a 'group' is an analysis of identical markers).

☒ All plots are contour plots with outliers or pseudocolor plots.

☒ A numerical value for number of cells or percentage (with statistics) is provided.

Methodology

Sample preparation	SH-SY5Y cells were inoculated into plates with densities of 1.5×105 cells per well and clutured overnight. Subsequently, cells were incubated with various nanoformulations for 6 h and then treated with 2 mM MPP+ for 24 h, after which the cells were harvested and dyed using Annexin V-FITC/PI apoptosis detection kit referred to the manufacture's protocol (Bestbio, Shanghai, China).
Instrument	Flow cytometer (Accuri C6, Beckman, NJ).
Software	All data obtained by flow cytometry were analyzed through the Beckman Coulter software.
Cell population abundance	In the parallel experiment, we selected the concentrated cell population as the target cell population, and selected the corresponding cell mass for subsequent analysis.
Gating strategy	We circle the gates by the degree of cell dispersion, and select the concentrated cell clusters for study.

☒ Tick this box to confirm that a figure exemplifying the gating strategy is provided in the Supplementary Information.
